# Phenotypic and Genomic Characterization of Nine String-Positive Carbapenem-Resistant Acinetobacter baumannii Isolates from Israel

**DOI:** 10.1128/spectrum.03002-22

**Published:** 2023-01-31

**Authors:** Nadya Rakovitsky, Mor N. Lurie-Weinberger, Amichay Hameir, Liat Wulffhart, Alona Keren Paz, David Schwartz, Yehuda Carmeli

**Affiliations:** a National Institute for Antibiotic Resistance and Infection Control, Israel Ministry of Health, Tel Aviv, Israel; b Sackler School of Medicine, Tel Aviv University, Tel Aviv, Israel; Emory University School of Medicine

**Keywords:** *A. baumannii*, string test positive, CRAB, antibiotic resistance, virulence

## Abstract

A positive “string test” indicates the ability of bacterial colonies grown on agar plates to form viscous strings of >5 mm when stretched. This phenotype is strongly associated with hypervirulence in Klebsiella pneumoniae but has never been described in carbapenem-resistant Acinetobacter baumannii (CRAB), an emerging human pathogen of high clinical significance. In this work, we screened 1,000 CRAB isolates, among which we identified and characterized 9 string-positive CRAB (stCRAB) isolates. Phenotypic and genotypic analyses revealed that the isolates were not phylogenetically related and possessed different antibiotic resistance and virulence profiles. Transmission electron microscopy (TEM) showed the presence of capsule in string-positive isolates. String-positive isolates were more motile but did not form more biofilm than non-string-positive isolates. They were less virulent in a murine thigh fitness model and a Galleria mellonella survival assay. In conclusion, here, we describe string-positive A. baumannii isolates and their phenotypic and molecular characteristics. We found that unlike K. pneumoniae, stCRAB isolates were not associated with increased virulence.

**IMPORTANCE** Acinetobacter baumannii has been considered a major health care threat in recent years. Despite many efforts, the pathogenesis and molecular mechanism of A. baumannii virulence remain poorly understood. Moreover, the plasticity of its genome frequently gives rise to new and more virulent isolates. Our current study is of significant importance as it concerns a previously undescribed A. baumannii phenotype. The string-positive phenotype is strongly associated with increased fitness and virulence in other Gram-negative bacteria such as K. pneumoniae. Although no clear correlation with virulence or fitness was found in our 9 stCRAB isolates, this could have been due to the limited statistical power of our research. We suggest that this phenotype should be taken into consideration as due to its genome plasticity, the next change can give rise to string-positive and hypervirulent strains, as is known for K. pneumoniae. Additional future research is needed regarding its possible consequences.

## INTRODUCTION

Acinetobacter baumannii is an important nosocomial pathogen, often multidrug resistant (MDR), that remains susceptible to only a few last-line antibiotic agents. It is listed by the U.S. Centers for Disease Control and Prevention as a serious threat to human health and by the World Health Organization as being at the highest priority for the development of and research into new drugs (1). Its genome plasticity has given rise to the MDR phenotype as well as a wide range of virulent strains.

The virulence of A. baumannii is not determined by a single virulence factor (2). Rather, it is multifactorial and is determined by the combination of multiple virulence factors. One of the most important virulence factors is the presence of the capsule (3). Among other virulence factors are outer membrane proteins (porins), biofilm production, quorum sensing, motility, as well as other factors (4). These factors play an important role in the pathogenicity of A. baumannii through their interactions with the host and competitors. However, determinants of A. baumannii virulence are not fully understood, and strains belonging to the same ST (sequence type) and carrying the same virulence gene contents may differ in their pathogenicity in a Galleria mellonella model (5).

We recently described mucoid carbapenem-resistant A. baumannii (CRAB) strains with elevated expression levels of the capsular gene *wzc*, that were associated with fulminant infections in patients and increased virulence in a G. mellonella model (6). We therefore paid special attention to the mucoid phenotype, and during routine screening of clinical samples, we observed an A. baumannii isolate with a “string test-positive” phenotype. This phenotype is defined as the ability of a bacterial colony to be stretched to a viscous filament (string) at least 5 mm from the surface of an agar plate (7). String test positivity has been reported previously for four bacteria, Vibrio cholerae, Aeromonas hydrophila, Pseudomonas aeruginosa, and Klebsiella pneumoniae (8), but was never described for A. baumannii. In K. pneumoniae, the string test-positive phenotype is highly correlated with hypermucoviscous phenotype and a hypervirulence phenotype (9). In East and Southeast Asian countries, this phenotype is associated with purulent infections (especially pyogenic liver abscess formation) and a high case fatality rate (10). Recently, the string test in K. pneumoniae was used for the prognosis of the virulence of strains (11). The molecular mechanisms of hypervirulence in hvKp (hypervirulent K. pneumoniae) have not been fully elucidated; however, they are associated with capsular polysaccharide (CPS) and exopolysaccharide (EPS) pathway genes in K. pneumoniae and the genes regulating them (i.e., the *rmpA*, *rmpA2*, and *magA* genes) (12). In A. baumannii, the capsule, composed of polyoligosaccharide units, glycoproteins, and lipooligosaccharides (LOSs), which form a shield around the bacteria, provides protection from the environment and is considered a virulence factor (13, 14).

The aim of this study was to determine the prevalence of the string test-positive phenotype among carbapenem-resistant A. baumannii isolates. Since string test-positive A. baumannii (stCRAB) has not been described previously, we also aimed to determine if the string phenotype is related to capsular types or represents EPS and if it is associated with biofilm production and motility, and we aimed to determine the antimicrobial resistance patterns, genetic relatedness, and virulence of the stCRAB isolates.

## RESULTS

### Prevalence and phenotypic characterization.

**FIG 1:**
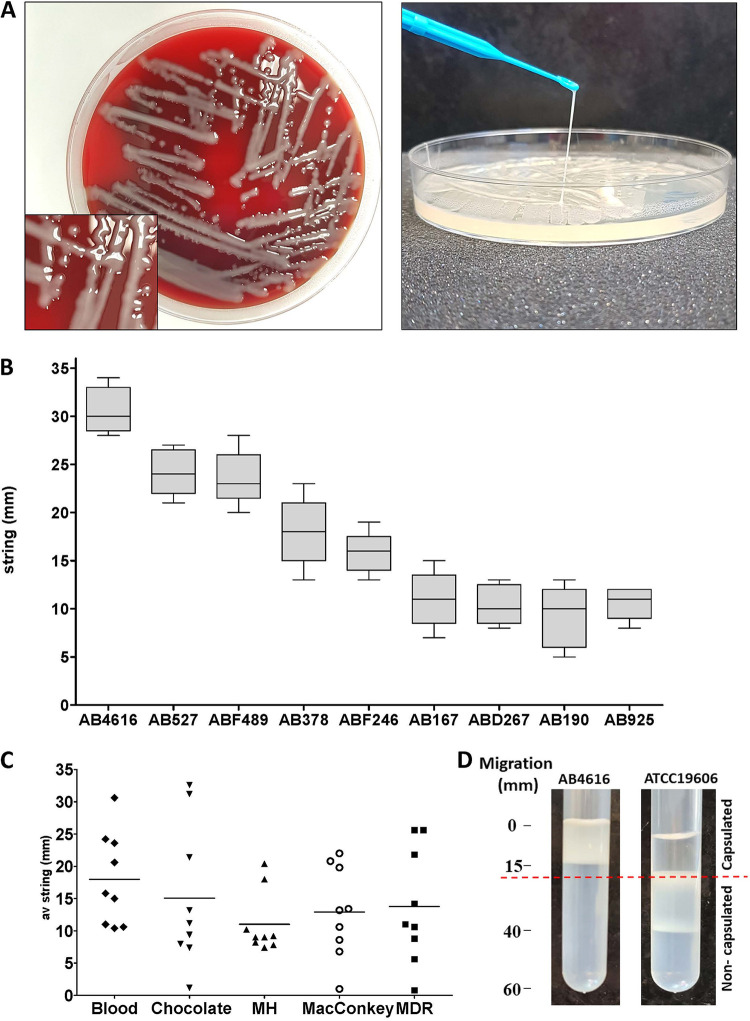
String test phenotypes in a number of clinical CRAB isolates. (A) Phenotype of the selected strain on a blood agar plate. (B) String measurement on a blood agar plate. At least 10 measurements from two plates were taken for each isolate. (C) Comparison of different media. Each dot represents a different strain. At least 10 measurements from two plates were taken for each strain. (D) Sample from the density gradient assay. The case isolate AB4616 migrated only to the top phase, indicating a capsulated bacterium. The control strain ATCC 19606 migrated to the bottom phase, indicating a thinly capsulated bacterium. Additional control strains can be seen in Fig. S1 in the supplemental material.

**FIG 2:**
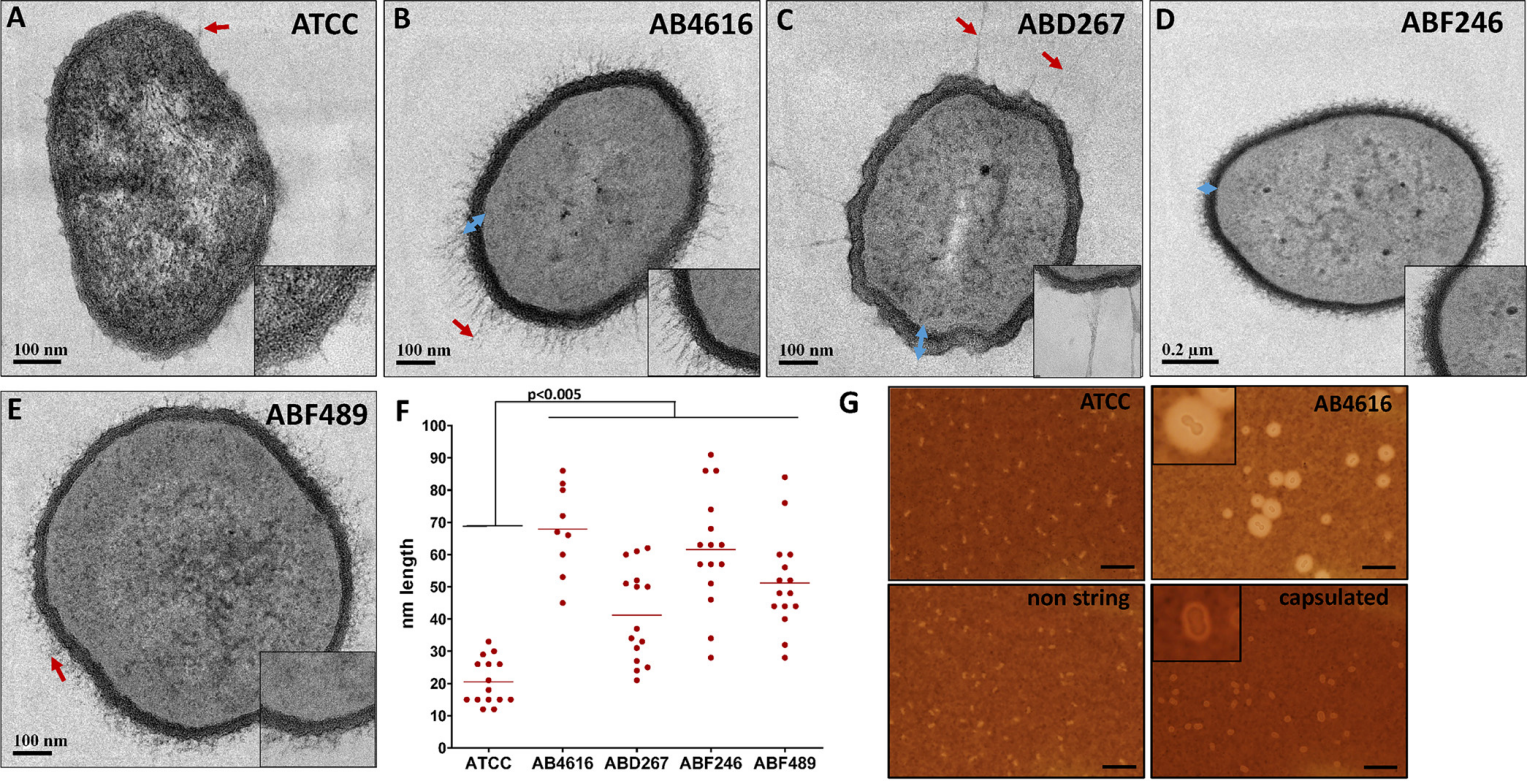
String-positive isolates produce capsule. (A to E) Representative TEM images of the string-positive isolates (AB4616, ABD267, ABF246, and ABF489) and ATCC 19606. Red arrows indicate bacterial filamentous extensions. Blue arrows indicate examples of measurements of the capsules. Insets show enlargements of the bacterial cell wall, filamentous extensions, and capsule. (F) Quantification of capsules measured in 15 places in 5 cells, presented in a graph. (G) India ink staining and representative capture of ATCC 19606, AB4616 (string isolate), a capsulated isolate, and a non-string-positive isolate (1 of the 12 control non-string-positive isolates). Bars, 10 μm. Insets show enlarged views of representative bacteria for AB4161 and the capsulated isolate.

We screened 1,000 CRAB isolates and identified 9 isolates that were string positive (stCRAB); i.e., they formed a viscous string of >5 mm when colonies on an agar plate were stretched (Fig. 1A). Thus, the prevalence of the stCRAB phenotype was 0.9% (95% confidence interval [CI], 0.0041 to 0.017). The length of the filamentous string varied among isolates and ranged between 9.2 ± 3.2 mm and 32 ± 2.4 mm (Fig. 1B). String formation was evident on both rich and selective agar plates, including blood, chocolate, Mueller-Hinton (MH), MacConkey, and MDR Acinetobacter agar plates (Fig. 1C; see also Table S1 in the supplemental material), but no significant difference was found among the different types of agar plates (*P* > 0.05). Seven of these isolates were clinical isolates from respiratory specimens, one was a screening isolate (from a patient skin sample), and one was from a hospital environmental culture (Table 1). As string phenotypes are associated with capsule production, we assessed whether the string-positive isolates were capsulated by a density gradient assay. All stCRAB isolates migrated to the top phase of a density gradient (Fig. 1D), similar to a capsulated control isolate (Fig. S1), indicating that they are large cells, possibly forming a capsule; all 12 non-stCRAB isolates migrated to the lower phase of the density gradient (Fig. S1), suggesting thin/noncapsulated bacteria. To visualize the capsule by transmission electron microscopy (TEM) imaging, we selected four isolates (AB4616, ABD267, ABF246, and ABF489); TEM revealed that the cells form a capsule with filamentous protrusions, with the diameter of the capsule ranging between 61.60 ± 18.06 nm (ABF489) and 41.20 ± 14.53 nm (ABD267). The thickest capsule was observed for isolate AB4616 (67.89 ± 13.71 nm). The capsule was 2- to 3-fold thicker than the capsule of the control strain ATCC 19606 (20.53 ± 7.13 nm) (Fig. 2A to F). In order to confirm the presence of capsule, India ink staining was performed on 9 stCRAB isolates and 12 non-string-positive CRAB strains (Fig. 2G); a thick capsule was observed for all stCRAB isolates, while all non-string-positive isolates had a very thin capsule around the cell, similar to ATCC 19606.

**TABLE 1:** Characteristics of the stCRAB isolates

Isolate	Yr of isolation	Site of isolation	ST	KL type
ABF489	2016	Tracheal aspirate	ST2	KL6
AB527	2020	Tracheal aspirate	ST2	KL9
AB4616	2019	Sputum	ST3	KL17
AB167	2019	Sputum	ST3	KL17
ABD267	2016	Sputum	ST3	KL17
AB925	2019	Skin	ST3	KL17
ABF246	2016	Tracheal aspirate	ST2	KL49
AB190	2019	Environment	ST3	KL17
AB378	2020	Tracheal aspirate	ST3	KL17

**FIG 3:**
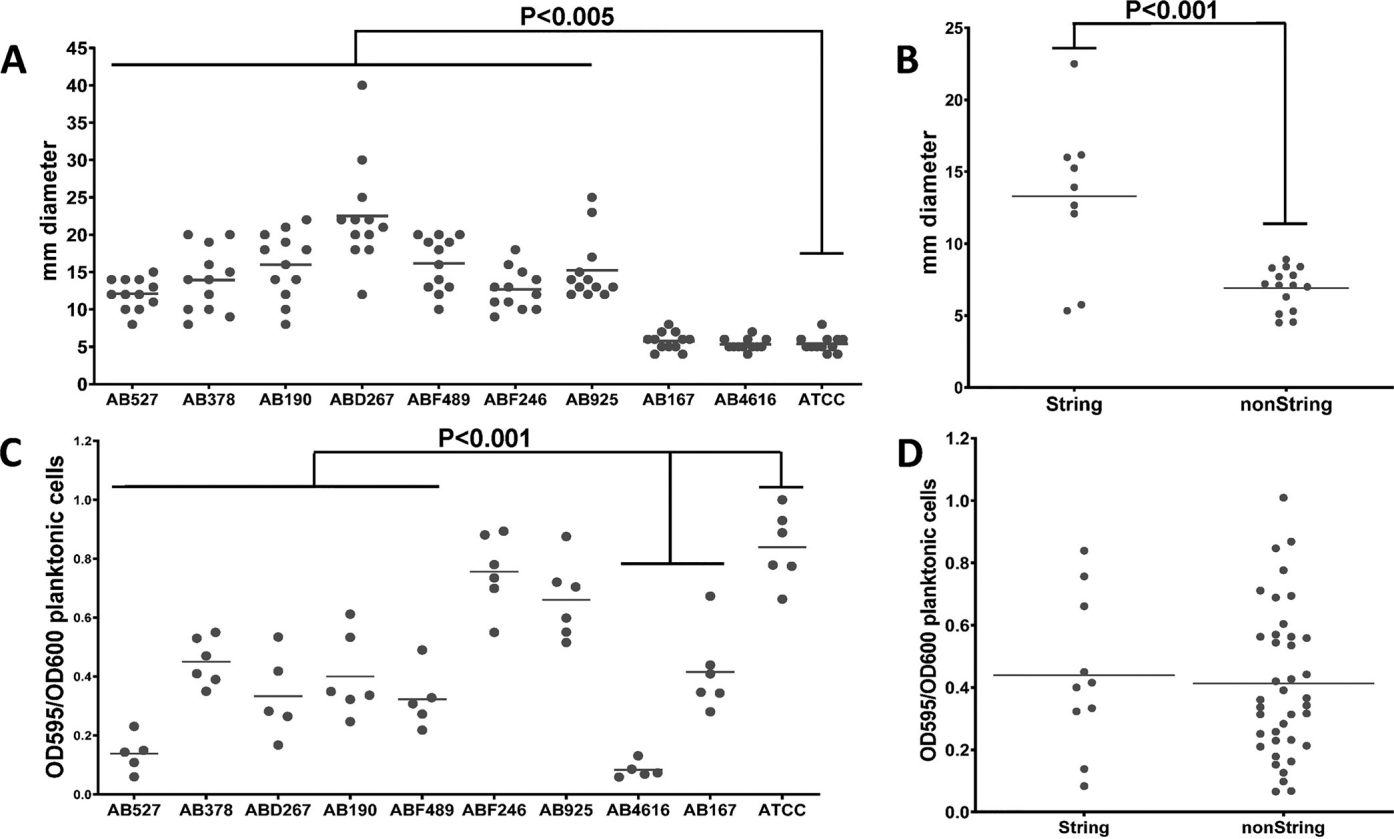
Phenotypic description of the study isolates. (A) Motility of string-positive isolates on Difco semisolid agar. Motility was calculated as the diameter of colony spread. ATCC 19606 was used as the control strain. Each dot represents the average measurement from a different plate. Medians are indicated by the lines. The *P* value was calculated using Student’s *t* test. (B) Motility of string-positive isolates analyzed in comparison to 12 randomly chosen clinical non-string-positive isolates. (C) Determination of the biofilm biomass formed by string-positive isolates and ATCC 19606 using a microtiter static biofilm model. The biofilm biomass was quantified following 18 ± 2 h of incubation at 37°C. Each isolate was tested in 6 replicates in 3 different experiments. Statistically significant decreases in biomass between the ATCC 19606 control strain and isolates AB527, AB378, ABD267, AB190, ABF49, AB4616, and AB167 are indicated. Medians are indicated by the lines. The *P* value was calculated using Student’s *t* test. (D) Biofilm biomass of string-positive isolates compared to the biofilm biomass of 12 randomly chosen clinical non-string-positive strains. Medians are indicated by the lines.

Lipooligosaccharides (LOSs) are involved in surface-associated motility and biofilm formation in Acinetobacter baumannii (15). So next, we tested surface-associated motility and biofilm formation. The measured motility range for all isolates was 4 to 40 nm, with 7/9 stCRAB isolates being highly motile, with an average diameter of motility on semisolid media of 22.5 ± 6.9 mm (range, 12 to 40 mm) (Fig. 3A), while 2 isolates (AB167 and AB4616) and the ATCC 19606 control strain were nonmotile, with an average growth diameter of 5 mm (individual average motility) (Table S2). The stCRAB isolates were significantly (*P* < 0.001) more motile than the control non-string-positive CRAB isolates (Fig. 3B). Seven of the stCRAB isolates (ABFF489, AB527, AB4616, AB167, ABD267, AB190, and AB378) formed significantly less biofilm (*P* < 0.001) than ATCC 19606 (Fig. 3C); however, as a group, the nine stCRAB isolates did not differ (*P* > 0.001) from the non-string-positive isolates in biofilm formation (Fig. 3D). None of the string test-positive isolates formed an air-liquid interface (ALI) biofilm (data not shown).

### Relatedness of the isolates and virulence determinants.

**FIG 4:**
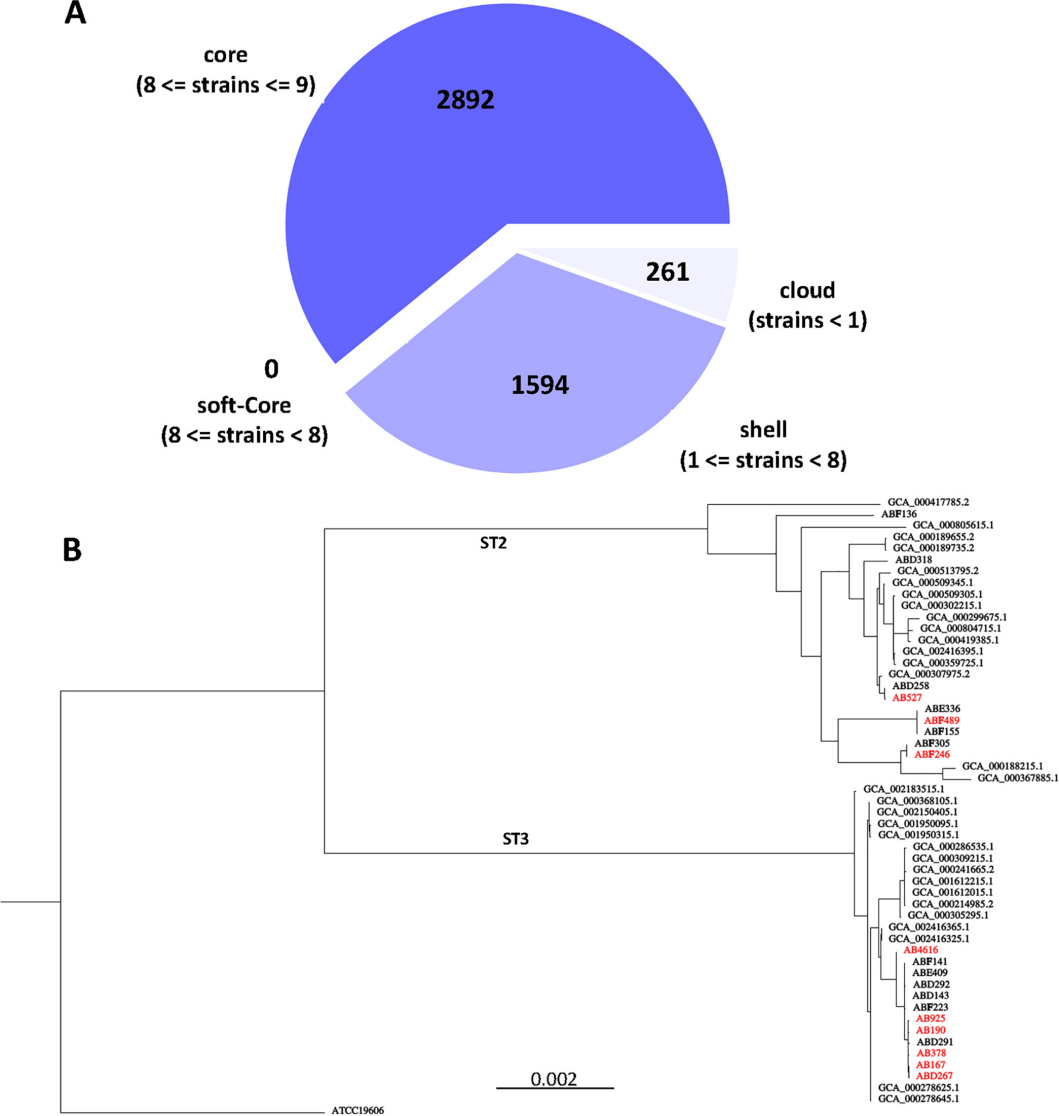
Genomic analysis of the string-positive isolates. (A) Venn diagram of the pangenome analysis of the 9 “string-positive” isolates and 12 non-string-positive isolates. (B) Core-genome alignment of the nine string-positive study isolates compared with randomly chosen publicly available CRAB genomes and ATCC 19606. String-positive isolates are indicated in red.

The complete genomes of the nine stCRAB isolates were obtained. Three strains belonged to Pasteur scheme sequence type 2 (ST2), and six belonged to ST3. The isolates belonging to the ST2 branch were ABF246, AB527, and ABF489, while ABD267, AB4616, AB190, AB167, AB925, and AB378 belonged to the ST3 branch. The ST2 isolates differed in capsule locus types, KL6, KL9, and KL49, while all ST3 strains shared the same capsule locus type (Table 1). Pangenome analysis of the 9 string-positive isolates and 12 non-string-positive isolates (Fig. 4A) showed that the vast majority of genes are shared by all genomes (2,892 core genes and 1,594 shell genes), regardless of the string phenotype. A phylogenetic tree based on the pangenome analysis of the 9 string-positive isolates shows their clear separation into ST2 and ST3 branches (Fig. S2). Core-genome alignment of the string-positive isolates compared to randomly chosen ST2 and ST3 genomes from a publicly available library showed that ST3 stCRAB isolates were closely related to each other; however, each of the ST2 stCRAB isolates belonged to a different branch on the ST2 phylogenetic tree (Fig. 4B; accession numbers are listed in Table S5). We also examined all nine stCRAB isolates by OrthoANIu (Table S3), which measures nucleotide-level genomic similarity between the coding regions of two genomes. We found two very closely related pairs, AB190 and AB167 (both ST2) and ABD267 and AB925 (both ST2), scoring 99.98% and 99.8%, respectively (Table S2). However, very high similarity, according to average nucleotide identity (ANI), was observed between isolates AB489 (ST3) and AB925 (ST2). Additionally, we performed single nucleotide polymorphism (SNP) distance analysis for the pairs of closely clustered isolates (Table S4). The SNP distances varied between 51 and 412 SNPs.

**FIG 5:**
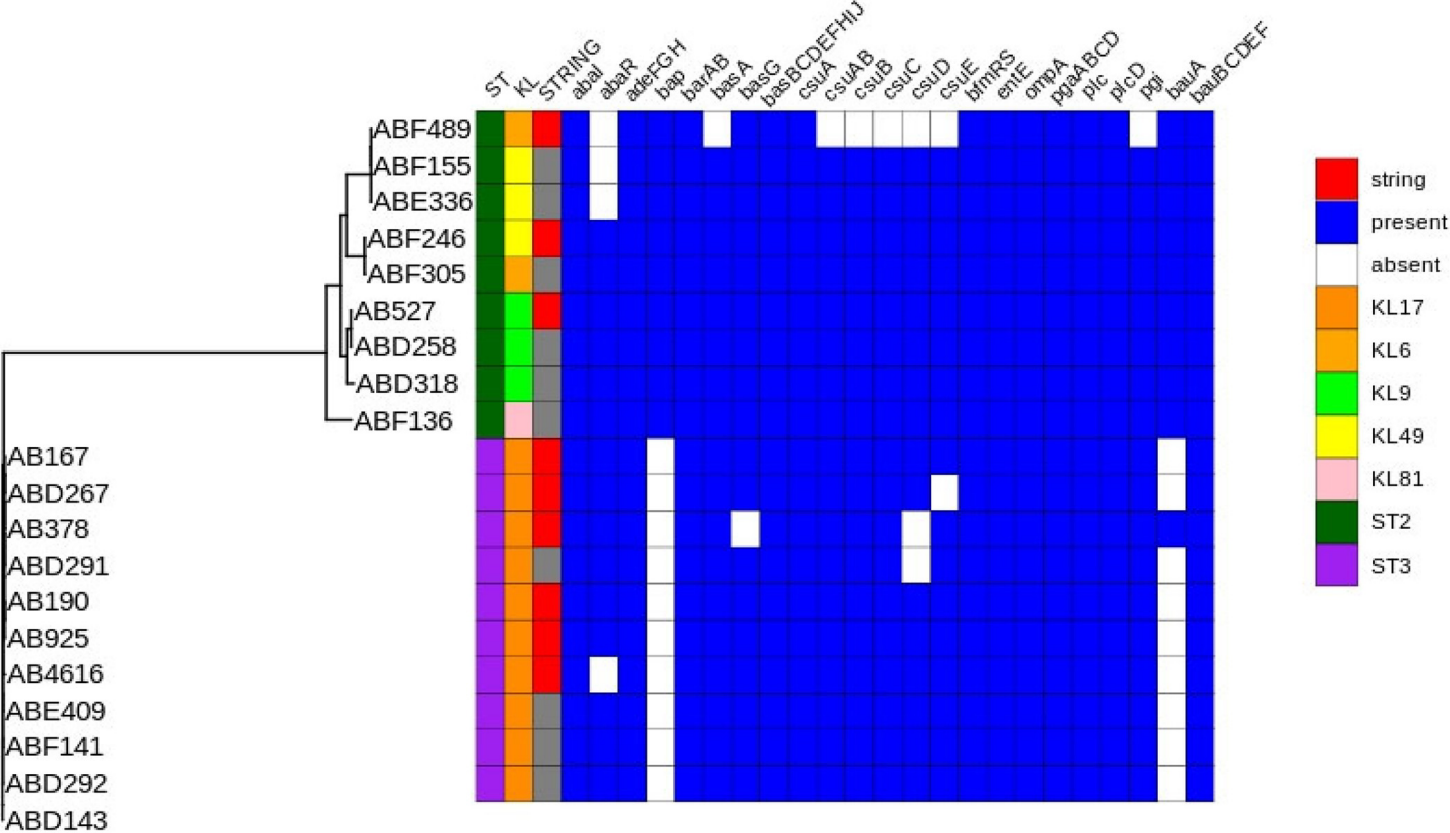
Virulome analysis of string-positive CRAB isolates collected in this study and non-string-positive clinical CRAB isolates. STs and KL types are included. The comparison of the genomes to the data in the Virulence Factor Database (VFDB) was complete; the existence of genes is represented in blue, and the absence of genes is represented in white.

A comparison of the genomes to the data in the Virulence Factor Database (VFDB) showed that all 9 study isolates possessed 30 virulence genes (*bauBCDEF*, *plcD*, *plc*, *pgaABCD*, *ompA*, *entE*, *bfmRS*, *basBCDEFGHIJ*, *barAB*, *abaI*, and *adeFGH*). These genes are related to various virulence functions, including iron uptake, biofilm formation, adherence, capsule regulation, and quorum sensing (*abaI*). Several differences in genomic content were evident. The biofilm-associated gene *bap* was present in ST2 stCRAB isolates (ABF489, AB527, and ABF246) but not in ST3 stCRAB isolates. Additional biofilm-associated genes were partially missing in some of the isolates: *csuE* was missing from ABD267 and ABF489, *csuD* was missing from AB378 and ABF489, and *csuC*, *csuB*, and *csuAB* were missing from ABF489. The iron uptake gene *bauA* was missing from ABD267, AB4616, AB925, AB167, and AB190. AB4616 did not have the *abaR* (quorum sensing) gene, and AB378 did not have the iron uptake gene *basG* (Fig. 5; the accession numbers of all genomes can be found in Table S5).

### Antibiotic resistance.

All study isolates were extremely drug resistant (XDR), testing resistant (or intermediately resistant) to all tested agents except colistin and tigecycline. AB4616 was also susceptible to gentamicin, tobramycin, and trimethoprim-sulfamethoxazole (Table 2).

**TABLE 2:** Antibiotic resistance profiles of the string test-positive isolates^*a*^

Strain	MIC (μg/ML) (resistance phenotype)																
	MERO	AMIK	GEN	ATM	CIP	P/T4	AUGC	C/T	COL	FOT	TGC	SXT	TOB	TAZ	CZA	IMI	ETP
AB527	16 (R)	>32 (R)	>8 (R)	16 (R)	>2 (R)	>32/4 (R)	>64/2 (R)	16/4 (R)	2 (S)	>8 (R)	0.5 (S)	<1/19 (S)	>8 (R)	>16 (R)	8/4 (R)	>16 (R)	>2 (R)
AB489	>16 (R)	8 (I)	>8 (R)	>32 (R)	>2 (R)	>32/4 (R)	>64/2 (R)	>32/4 (R)	1 (S)	>8 (R)	1 (S)	>8/152 (R)	>8 (R)	>16 (R)	>16/4 (R)	16 (R)	>2 (R)
ABF246	>16 (R)	>32 (R)	>8 (R)	>32 (R)	>2 (R)	>32/4 (R)	>64/2 (R)	>32/4 (R)	1 (S)	>8 (R)	1 (S)	>8/152 (R)	>8 (R)	>16 (R)	>16/4 (R)	>16 (R)	>2 (R)
AB167	>16 (R)	8 (I)	>8 (R)	>32 (R)	>2 (R)	>32/4 (R)	>64/2 (R)	>32/4 (R)	2 (S)	>8 (R)	1 (S)	>8/152 (R)	8 (R)	>16 (R)	>16/4 (R)	16 (R)	>2 (R)
AB190	>16 (R)	>32 (R)	>8 (R)	16 (R)	>2 (R)	>32/4 (R)	>64/2 (R)	16/4 (R)	0.5 (S)	>8 (R)	0.5 (S)	>8/152 (R)	>8 (R)	>16 (R)	8/4 (R)	8 (R)	>2 (R)
AB378	>16 (R)	>32 (R)	>8 (R)	>32 (R)	>2 (R)	>32/4 (R)	>64/2 (R)	>32/4 (R)	0.5 (S)	>8 (R)	0.5 (S)	>8/152 (R)	8 (R)	>16 (R)	>16/4 (R)	>16 (R)	>2 (R)
AB925	4 (I)	8 (I)	>8 (R)	>32 (R)	>2 (R)	>32/4 (R)	>64/2 (R)	16/4 (R)	1 (S)	4 (R)	0.5 (S)	>8/152 (R)	4 (I)	>16 (R)	>1/4 (R)	>16 (R)	>2 (R)
AB4616	16 (R)	8 (I)	0.5 (S)	>32 (R)	>2 (R)	>32/4 (R)	>64/2 (R)	>32/4 (R)	1 (S)	>8 (R)	0.5 (S)	<1/19 (S)	1 (S)	>16 (R)	>16/4 (R)	4 (I)	>2 (R)
ABD267	>16 (R)	4 (S)	>8 (R)	>32 (R)	>2 (R)	>32/4 (R)	>64/2 (R)	>32/4 (R)	2 (S)	>8 (R)	1 (S)	>8/152 (R)	>8 (R)	>16 (R)	>16/4 (R)	16 (R)	>2 (R)

a MERO, meropenem; AMIK, amikacin; GEN, gentamicin; ATM, aztreonam; CIP, ciprofloxacin; P/T4, piperacillin/tazobactam constant 4; AUGC, amoxicillin/clavulanic acid constant 2; C/T, ceftolozane-tazobactam; COL, colistin; FOT, cefotaxime; TGC, tigecycline; SXT, trimethoprim-sulfamethoxazole; TOB, tobramycin; TAZ, tazobactam; CZA, ceftazidime-avibactam; IMI, imipenem; ETP, ertapenem; R, resistant; S, susceptible; I, intermediately resistant.

Genomic analysis showed that all stCRAB isolates carry a *bla*_OXA-23_ carbapenemase gene, a *bla*_ADC-6_ *ampC* cephalosporinase gene, and the intrinsic *bla*_OXA-51_ gene. All ST2 isolates shared *aph(3′)-Ia*, and all ST2 isolates and one ST3 isolate (AB378) shared *aph(6)-Id*, *aph(3′)-Ib*, and the macrolide resistance genes *msr*(E) and *mph*(E). The complete resistome of the characterized isolates is presented in Table S6.

### *In vitro* and *in vivo* fitness and virulence.

**FIG 6:**
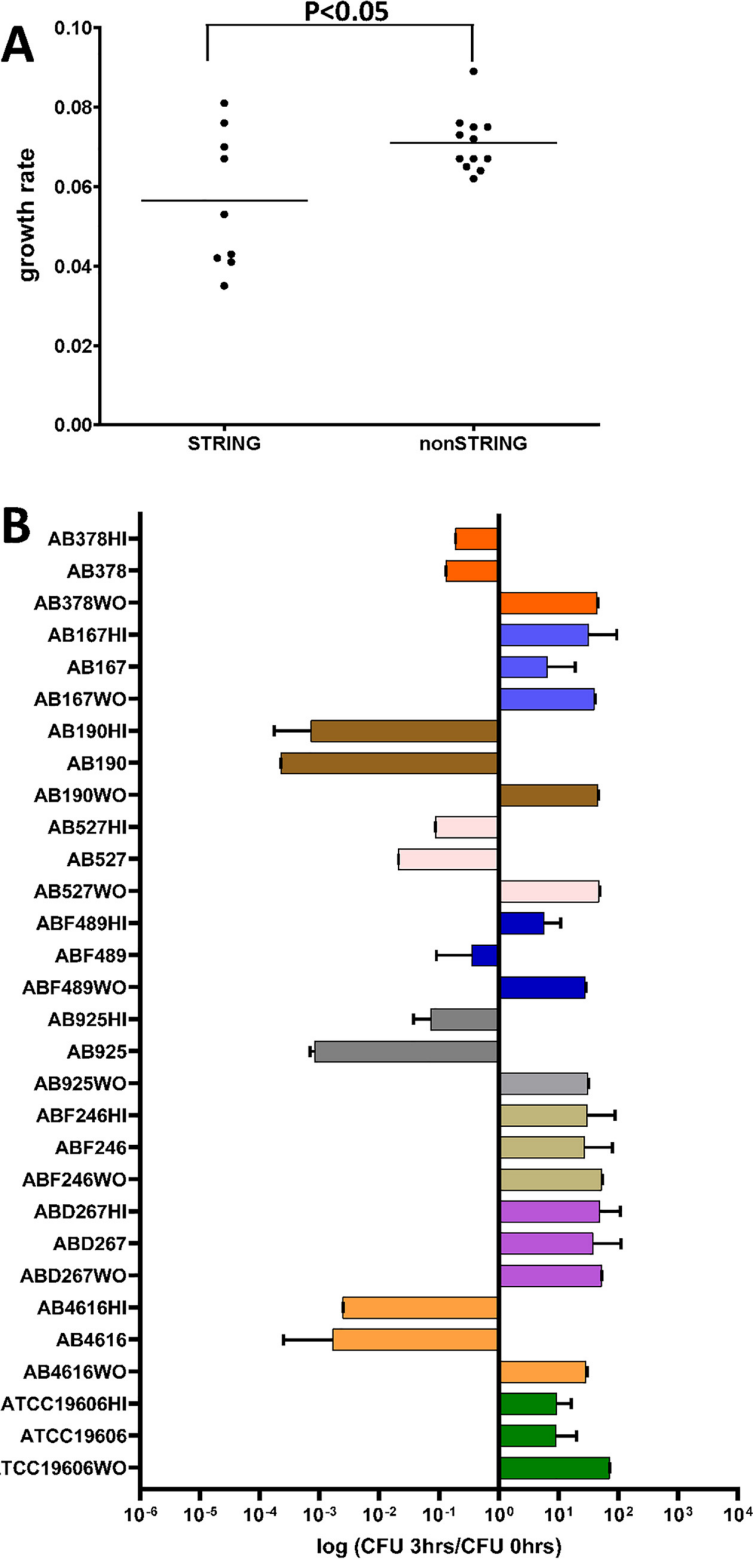
*In vivo* fitness ability and survival in serum. (A) Growth rate analysis of string-positive isolates versus non-string-positive isolates. Means and SDs were calculated for 9 isolates, in five replicates. Student’s *t* test was performed (*P* < 0.05). (B) Survival of string-positive A. baumannii isolates in 80% NHS and heat-inactivated NHS (as a control for each strain grown [WO] in BHI medium measured). The data presented are normalized to CFU counts at time zero. Each bar indicates the mean ± SD from at least three independent experiments. WO, without serum. *, *P* < 0.05 compared with ATCC 19606 by Student’s *t* test.

We analyzed the *in vitro* growth rates of the 9 stCRAB isolates (Fig. S3) and compared them to those of 12 randomly chosen clinical isolates with the non-string-positive phenotype. We found that the growth rates of the stCRAB isolates varied more than those of the control group isolates. stCRAB isolates, as a group, had lower growth rates (0.056 ± 0.017 versus 0.071 ± 0.007 [*P* < 0.05]) (Fig. 6A), longer doubling times, and lower maximum growth (Table S7) than the controls. There was no difference in lag times between the string-positive and non-string-positive groups. Compared to the non-string-positive isolates, the stCRAB isolates have a growth defect, and this is not surprising due to the fitness cost of producing a large capsule.

We analyzed *in vitro* virulence by testing the survival of bacteria in pooled normal human serum (NHS) or heat-inactivated normal human serum (NHS-HI) (Fig. 6B). We found that stCRAB isolates behave heterogeneously in serum. ABF489 exhibits resistance to killing by complement; to some extent, a similar effect can be seen for AB925. Isolates AB925, ABD267, AB378, and AB167 were serum resistant; on the other hand, isolates AB4616, AB925, AB527, and AB190 were serum sensitive. We did not find a correlation with the ST or the length of the string.

**FIG 7:**
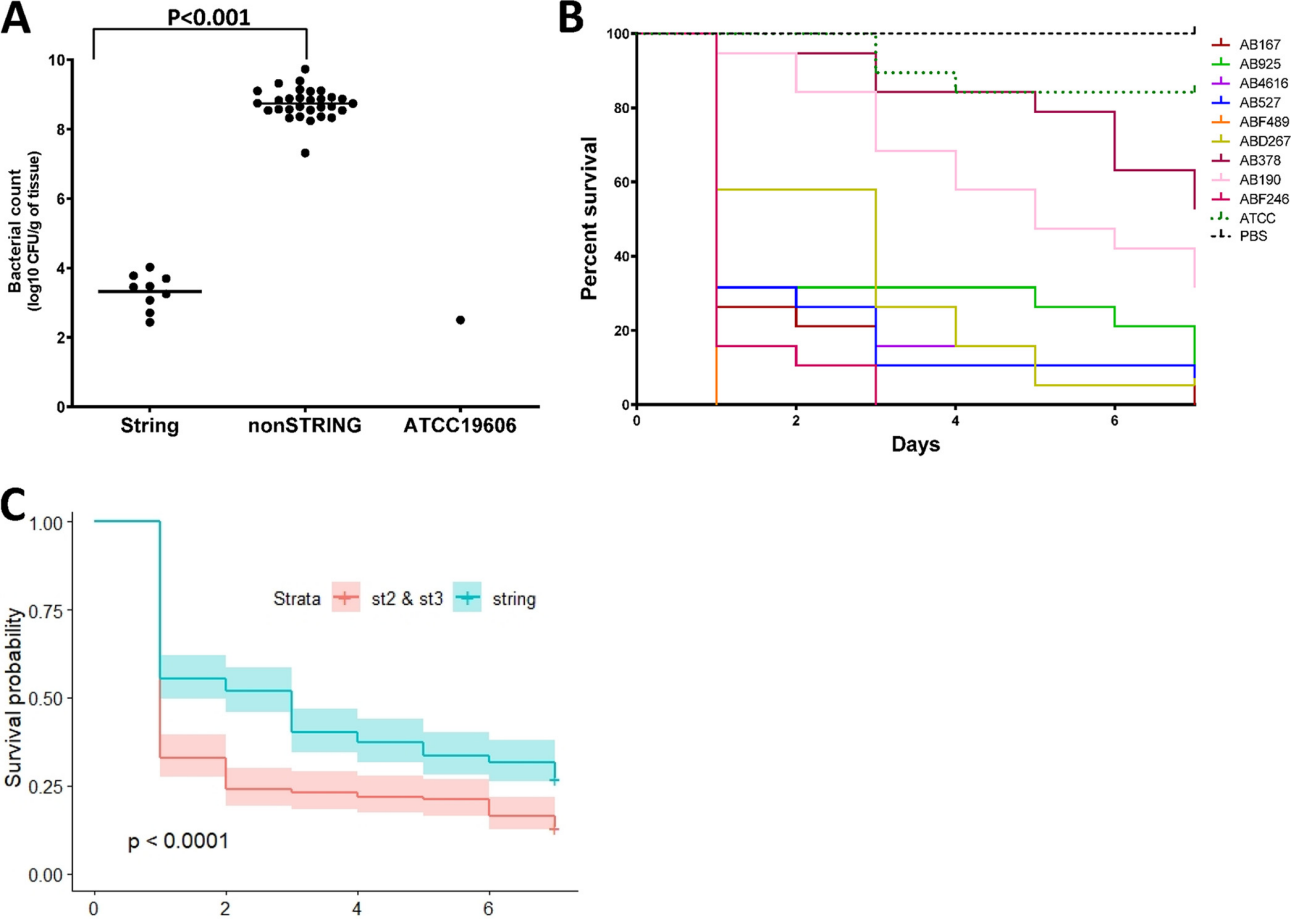
*In vivo* models. (A) Determination of bacterial loads in the thighs of mice infected with A. baumannii isolates (*n* = 5 per group). The bacterial load was significantly lower in all string-positive isolates than in non-string-positive clinical isolates (*P* < 0.001). Each dot represents the mean for 5 mice. Student’s *t* test was performed (*P* < 0.001). (B) Kaplan-Meier survival curves for G. mellonella larvae (*n* = 20 per group) infected with 1 × 10^5^ CFU of each strain of A. baumannii. Curves were compared via the Mantel-Cox test with the Bonferroni correction for multiple comparisons. ATCC 19606 showed significantly increased survival compared to the string test-positive isolates. (C) Kaplan-Meier survival curves for G. mellonella larvae (*n* = 180 per group) infected with 1 × 10^5^ CFU of string-positive isolates of A. baumannii versus non-string-positive clinical A. baumannii isolates. The probability of survival for non-string-positive clinical isolates is lower than that for stCRAB isolates.

We used two *in vivo* models to assess virulence. The first *in vivo* model used was a murine thigh infection model where bacterial fitness was tested 24 h after injection. The average bacterial count in mice injected with stCRAB isolates was similar to that of the ATCC 19606 control (3.32 ± 0.67 versus 2.47 ± 0.57 log_10_ CFU/g tissue [*P* ≥ 0.1]) (Fig. 7A). Compared to clinical CRAB isolates, the average bacterial count of the stCRAB isolates was low (3.32 ± 0.67 log_10_ CFU/g tissue versus 8.67 ± 0.64 log_10_ CFU/g tissue for virulent clinical isolates [*P* ≤ 0.005]) (Fig. 7A).

The second *in vivo* model was the G. mellonella survival model, which revealed that the rate of survival of G. mellonella larvae varied between 5% and 95% after injection; AFF489 and ABF246 were the most virulent (mortality rates of 80% and 95%, respectively) (Fig. 7B). Interestingly, the median survival rate of larvae injected with stCRAB isolates was significantly (*P* < 0.005) higher than that of larvae infected with non-string-positive control isolates (Fig. 7C).

## DISCUSSION

The high plasticity of the CRAB genome could potentially lead to the emergence of novel resistant and virulent isolates, and thus, monitoring new CRAB phenotypes is highly important. CRAB species can form mucoid colonies (6), and two recent studies linked A. baumannii mucosity and capsule formation to the clinical severity and epidemic potential of the strains (6, 16). A. baumannii can undergo a phase variation switch from a matte to an opaque phenotype with different virulence characteristics (17). In K. pneumoniae, isolates with a string-positive phenotype are known to be hypervirulent (9); until now, this phenotype was not described in A. baumannii. Here, we first describe a string-positive phenotype in A. baumannii. We determined that this phenotype is rare, found among 9 out of 1,000 CRAB isolates in Israel. In this study, we characterize these 9 string-positive CRAB isolates and explore the correlations among string phenotype and genotype, capsule production, biofilm formation, motility, growth rate, and fitness in a mouse model and in serum and survival in a G. mellonella model.

The isolates in this study belonged to the two most common CRAB sequence types (STs) in Israel: ST2 and ST3 (18, 19). The ST3 isolates were closely related to each other phylogenetically. Indeed, core-genome analysis suggests that ABF489 evolved into ABF246 while acquiring more virulence genes. The ST2 isolates were not grouped phylogenetically. Whole-genome sequencing (WGS) and antibiotic resistance profiling revealed that all isolates harbored different sets of resistance genes and displayed different resistance phenotypes. These results imply that the string test-positive phenotype occurs in CRAB isolates with various evolutionary origins.

Plasmids carrying virulence genes have been described in hypervirulent K. pneumoniae isolates (20, 21). However, we did not find any plasmids in string-positive CRAB isolates. However, according to TEM imaging, India ink staining, and density gradient assays, all string-positive CRAB isolates produce capsules larger than those produced by non-string-positive isolates or ATCC 19606.

The association between the string test-positive phenotype and static biofilm formation in hypervirulent K. pneumoniae is not clear. However, Cubero et al. (22) recently showed an association between hypervirulent K. pneumoniae serotype K1 clinical isolates and the formation of ALI biofilms. An air-liquid interface biofilm is actually a perfect ecological niche for bacteria because it provides some nutrients from the medium and oxygen from the air. This has already been described in pathogenic bacteria, including A. baumannii (23). In our study, the stCRAB isolates did not form floating biofilms. In addition, the string test-positive isolates formed less-static biofilms than randomly chosen control isolates. Interestingly, *bap*, the best-characterized biofilm-associated gene in A. baumannii (24), did not differ between biofilm-producing and non-biofilm-producing stCRAB isolates, suggesting that this is not the gene directly responsible for the phenotypic difference. Instead, the *bap* gene was absent from all ST3 string-positive isolates and present in all ST2 string-positive isolates, likely reflecting their divergent evolutionary histories. We observed variation in the presence of additional biofilm-associated genes in several isolates, and the low level of biofilm formation by ABF489 is likely associated with the absence of *csuE*, *csuD*, *csuC*, *csuB*, and *csuAB*.

We found that seven of the nine stCRAB isolates were more motile than the control strain ATCC 19606 and the randomly chosen control clinical strains. These results are complementary to the results on biofilm formation since biofilm formation and motility are usually mutually exclusive lifestyles in bacterial populations (25). The bacterial capsule plays a role in protecting bacteria from the environment, such as the host immune response, and serum resistance may serve as a marker of Acinetobacter virulence (26–28). However, as with other virulence-related phenotypes, the stCRAB isolates that we tested behaved heterogeneously.

We did not find evidence that stCRAB isolates had higher *in vivo* fitness than the non-string-positive controls. In the murine thigh infection model, string-positive isolates displayed fitness similar to that of the control nonvirulent strain but lower fitness than that of virulent clinical isolates. In the G. mellonella survival model, we saw similar results: the string-positive strains were more virulent than the control nonvirulent strain (ATCC 19606) but less virulent than the non-string-positive clinical isolates.

Interestingly, we found an inverse relationship between biofilm formation and virulence. It is believed that biofilm formation by A. baumannii contributes to its virulence. Indeed, virulent strains generate more biofilms than less virulent strains (29). However, in our study, the most virulent isolates in the G. mellonella model were AB167, ABF246, and ABF489, only one of which (ABF246) showed a high biofilm formation score.

Since this is the first observation of this phenotype, the screening of 1,000 isolates and the detection of 9 stCRAB isolates allowed us to perform group-level analysis. Our results suggest that the string-positive phenotype in CRAB is not associated with increased virulence. This conclusion should be taken with caution as our study has limited statistical power to detect differences between groups, we did not have clinical correlates, and our isolates represent only one geographical area. The increasing virulence of A. baumannii (30) and its clinical success, which is attributed to its plastic genome and high capacity to acquire new genetic determinants (14), highlight the necessity of surveillance focused on this newly increasing phenotype.

In conclusion, unlike in the case of K. pneumoniae, the string-positive phenotype in CRAB was not correlated with increased fitness and virulence.

## MATERIALS AND METHODS

### Description of the isolates.

The study isolates are part of a large collection of carbapenem-resistant Acinetobacter baumannii (CRAB) isolates. This collection includes over 10,000 isolates from hospitals and long-term-care facilities in Israel isolated between 2004 and 2021. Following the fortuitous detection of a string test-positive isolate, we performed systemic screening of 1,000 isolates from this collection for this phenotype. The study isolates were identified using the Vitek 2 system (bioMérieux, Marcy-l’Etoile, France), followed by confirmation of the presence of *bla*_OXA-51_. Antibiotic susceptibility was determined using broth microdilution (Sensititre GN6F plate; Thermo Fisher Scientific, Oakwood Village, OH, USA), according to CLSI M07 guidelines (31), for all string-positive samples. The controls were ATCC 19606 and 12 non-string-positive isolates, 6 of ST2 and 6 of ST3 (Pasteur scheme), the most common sequence types in Israel. These controls were chosen at random from the laboratory’s collection.

### Phenotypic characterization.

**(i) String test.** Isolates were grown on TSA (tryptic soy agar) blood agar plates overnight at 37°C and stretched using an inoculation loop. The string was manually measured with a ruler. Isolates that produced colonies that could be stretched into a viscous string of >5 mm in all cases tested were considered string test positive. At least 15 different colonies of each suspected isolate were measured in 3 independent experiments. Bacterial isolates were also grown on blood, chocolate, Mueller-Hinton (MH), MacConkey, and MDR Acinetobacter agar plates (HyLabs, Rehovot, Israel) overnight at 37°C.

**(ii) India ink staining.** The India ink staining method described previously by Bachman et al. (32) was used. In brief, isolates were grown overnight at 37°C in LB. On the following day, a small drop (10 μL) of bacteria was placed onto a slide and air dried for 30 min. Dried bacteria were fixed to the slide for 3 min at 56°C. A small drop of India ink stain (diluted 1:3 with phosphate-buffered saline [PBS]) was placed onto the slide and covered with a coverslip. Slides were viewed with an Olympus BX41 bright-field microscope and imaged with Olympus cellSens Entry v1.9 software.

**(iii) Density gradient.** The sizes of the study isolates were tested by a density-dependent gradient test (33). Isolates were grown overnight at 37°C either on TSA blood agar plates or in BHI (brain heart infusion) medium with shaking. In brief, a matrix with a volume of 2 mL, composed of silica at concentrations of 30% (vol/vol) for the bottom phase (1 mL) and 50% for the top phase (1 mL), was prepared. After the addition of the bacterial inoculum, the tube was centrifuged for 30 min at 3,000 × *g*. Based on inspection, a bacterial band that migrated to the bottom phase of the gradient was classified as not capsulated or thinly capsulated. A bacterial band that was mostly at the top phase was classified as capsulated.

**(iv) Transmission electron microscopy.** Transmission electron microscopy (TEM) analysis was undertaken at the Institute for Nanotechnology and Advanced Materials (Bar Ilan University, Israel). Isolates were grown overnight at 37°C on TSA blood agar plates. The samples were prepared according to a standard protocol (34) and as described previously (6). The samples were fixed for 3 h in Karnovsky fixative (2.5% glutaraldehyde with 2.5% paraformaldehyde in 0.1 M sodium cacodylate buffer [pH 7.4]) and then washed with 0.1 M sodium cacodylate buffer. The cells were postfixed in a solution containing 1% OsO_4_, 0.5% K_2_Cr_2_O_7_, and 0.5% K_4_[Fe(CN)_6_] in 0.1 M cacodylate buffer (pH 7.4) for 1 h at room temperature and then washed twice with 0.1 M cacodylate buffer, followed by rising with double-distilled water (ddH_2_O) three times. Bacterial cells were then stained with 2% uranyl acetate for 1 h, washed with ddH_2_O, dehydrated in ethanol, and embedded in Epon Embed 812 resin (Electron Microscopy Sciences [EMS]). The resin was polymerized at 60°C for 24 h. Ultrathin sections (90 to 70 nm) were obtained with a Leica ultracut (UC7) ultramicrotome and then analyzed with an FEI G-12 Spirit electron microscope. The statistical significances of the differences between the string isolates and the control ATCC 19606 strain were determined using one-way analysis of variance (ANOVA) in GraphPad Prism 9.3.1.

**(v) Planktonic growth.** Cultures grown overnight in BHI medium at 37°C with shaking were diluted 1:1,000 in medium (BHI medium). Aliquots with a volume of 200 μL were inoculated into a 96-well plate (Nunc MicroWell 96-well microplates; Thermo Scientific), followed by incubation at 35°C ± 2°C with shaking (120 rpm) in the same medium. Growth was monitored by reading the optical density at 600 nm (OD_600_) every 30 min for 24 h (Synergy HT; BioTek, Agilent, Winooski, VT). The generation time, lag time, and maximal OD were calculated directly from the growth curve (35). Each experiment was performed in 5 replicates and repeated in 3 independent experiments. The statistical significances of the differences between the string- and non-string-positive groups were determined using Student’s *t* test in GraphPad Prism 9.3.1.

**(vi) Motility.** Cultures grown overnight in BHI medium at 37°C with shaking were diluted 1:1,000 in medium (BHI medium). The motility assay was performed on freshly prepared semisolid (0.25% agar) Difco agar (BD, USA) plates using a previously described protocol (6). The agar was stabbed with approximately 1 μL of a diluted bacterial suspension, followed by a 24-h incubation at 37°C. Surface motility was assessed by the measurement of the diameter of bacterial growth. Four experiments (quadruplicates per experiment) were performed for each isolate. A motile strain (AB077) and a nonmotile strain (ATCC 19606) were used as controls. The statistical significances of the differences between the string- and non-string-positive groups were determined using Student’s *t* test in GraphPad Prism 9.3.1.

**(vii) Biofilm studies.** Both static and air-liquid interface (ALI) biofilm formation were assessed.

For the assessment of the static biofilm biomass, cultures grown overnight in BHI medium at 37°C with shaking were diluted 1:1,000 in medium (BHI medium). Aliquots with a volume of 200 μL were inoculated into a 96-well plate (Nunc MicroWell 96-well microplates; Thermo Scientific), followed by incubation at 35°C ± 2°C without shaking in the same medium for 24 h. A biofilm assay was performed using 0.1% crystal violet staining and was fully described previously (5). Each isolate was tested in triplicates. Means and standard deviations (SDs) were calculated. The statistical significances of the differences between the string-positive group and the control groups were determined using a Mann-Whitney U test.

ALI biofilm production was studied using an ALI biofilm assay (36). Briefly, a single colony was inserted into 2 mL BHI medium in 12-mm-diameter polystyrene tubes, vortexed for 2 min, and incubated for 3 days at 37°C without shaking. The identification of ALI biofilm-producing isolates was done by visual examination.

**(viii) Serum resistance assay.** The recalcification method described previously by Moghaddam et al. (37) was used to prepare pooled human serum from at least 5 different people. Isolates were grown in BHI medium overnight at 37°C with shaking. The isolates were spun down, washed two times with PBS, and adjusted to a final concentration of 1 × 10^7^ CFU/mL with PBS. A total of 1 × 10^5^ CFU was added to 5 mL of 80% NHS (normal human serum) in PBS and 80% heat-inactivated NHS in PBS. The samples were incubated with shaking at 37°C for 0 and 3 h. Serial dilutions on TSA blood agar plates were done in order to count the surviving bacteria. Three experiments were done as biological replicates. The statistical significance of the differences between the results of the serum resistance assay was determined using Student’s *t* test in GraphPad Prism 9.3.1.

### WGS and bioinformatics analysis.

DNA extraction was performed on the Microlab Nimbus workstation (Hamilton, Reno, NV, USA) with a STARMag 96 universal kit (Seegene, Seoul, Republic of Korea). Samples D267, F246, and F489 were sequenced at the Sequencing Core at the University of Illinois, Chicago. Samples AB167, AB190, AB378, AB527, AB925, and AB4616 were sequenced at the Sequencing Core at Rush University, Chicago, IL. Libraries for both groups were prepared using the Nextera XT kit (Illumina Inc., CA, USA), followed by sequencing on the Illumina Novaseq platform (Illumina Inc.), and then assembled using Spades V3.12.0 using the high-output 2 × 150-bp kit (Illumina Inc.).

Isolate typing was performed by MLST (multilocus sequence typing) using the Pasteur scheme, using PubMLST (https://pubmlst.org). Capsular polysaccharide (KL) and lipooligosaccharide outer core (OCL) synthesis were determined using Kaptive version 0.5 (38).

Resistance genes were detected by comparing all open reading frames (ORFs) against the VFDB (Virulence Factor Database) (http://www.mgc.ac.cn/VFs/main.htm) and the CARD (Comprehensive Antibiotic Resistance Database) (39). *ampC* gene classification was performed against the PubMLST database (40).

Plasmids were detected based on BLASTn similarity and then divided into homology groups according to the parameters established previously by Carattoli et al. (DNA identity of 74% and coverage of 90%) (41). Isolates that matched more than one group were assigned to the GR group with the highest identity score. The wide-range *Enterobacteriaceae* plasmid was detected using PlasmidFinder version 2.0.1 (42).

Pangenome analysis of stCRAB genomes was done using Roary version 3.12.0 (43). The resulting core genes (ORFs found in 99% of the isolates) were aligned using RAxML version 8.2.12 (44) with the general time-reversible (GTR) gamma model. Next, 16 sequenced genomes belonging to ST2 and 16 genomes belonging to ST3 were added. These strains were chosen from the GTDB-Tk version 0.3.2 database. The strains were randomly selected from different laboratories and geographic locations to allow diversity in the pangenome analysis with Roary, and a full tree was again constructed using RAxML with the GTR gamma model. Their accession numbers and STs are presented in Table S5 in the supplemental material.

### *In vivo* models.

Two *in vivo* models were used: (i) a killing assay in Galleria mellonella and (ii) a neutropenic murine bacteremia and sepsis model.

### (i) G. mellonella killing assay.

Final-instar-stage research-grade G. mellonella larvae were obtained from TruLarv (Biosystems, Devon, UK). They were stored and used according to the manufacturer’s instructions. The G. mellonella killing assay was previously described by Rakovitsky et al. (6). In brief, bacteria were grown in 5 mL BHI medium overnight at 37°C with shaking, diluted to an OD of 0.1, grown for another 2 to 3 h until they reached logarithmic phase, diluted to 10^5^ cells in 20 μL, and injected with a 32-gauge needle (Hamilton, Reno, NV, USA) into larvae (20 larvae per group). Infected larvae were incubated for up to 7 days in a dark environment, and their viability was checked every 24 h. Kaplan-Meier survival curves were carried out (for individual-isolate survival [20 per group] and group survival [180 per group for all string-positive isolates and 240 per group for non-string-positive isolates]) using GraphPad Prism 9, and statistical significance was calculated using a log rank test.

### (ii) Murine bacteremia and sepsis model.

As previously described (44), pathogen-free female ICR mice (Envigo, IL), weighing about 22 to 25 g and 5 to 6 weeks of age, were used in all experiments. All animals were housed in individual cages under constant temperature (22°C) and humidity with a 12-h light/dark cycle and had access to food and water *ad libitum* throughout the study. Mice were rendered neutropenic by the intraperitoneal (i.p.) injection of 150 mg/kg of body weight of cyclophosphamide (Sigma, Rehovot, Israel) on day 4 and 100 mg/kg on day 1 preinfection. Bacteria were grown in 5 mL BHI medium overnight at 37°C with shaking, diluted with BHI medium to an OD of 0.1, grown at 37°C with shaking until they reached logarithmic phase, and diluted with saline solution to 10^6^ CFU in 100 μL. Thigh infection was induced by injecting 100 μL of 10^6^ CFU per mouse intramuscularly (into the thigh). The inoculum was diluted and plated onto TSA plates at 35°C ± 2°C overnight, followed by CFU reading 24 h later in order to count the CFU from the initial injection. To determine the thigh bacterial load, mice were sacrificed after 24 h, and the thigh was aseptically removed, weighed, and homogenized individually in 1 mL of saline solution. Diluted samples were plated onto TSA plates at 35°C ± 2°C overnight. The results are presented as the number of CFU per gram of infected thigh. Three independent experiments were done for each isolate (*n* = 5), and the results were averaged. Nonmanipulated mice (*n* = 5) and PBS-injected mice (*n* = 5) were used as controls. The protocol was approved by the Institutional Animal Care and Use Committee at Tel-Aviv Sourasky Medical Center.

### Data availability.

The data set presented in this study and the genome assemblies were uploaded to and are publicly available in the NCBI repository under BioProject accession number PRJNA849753 (AB167, BioSample accession number SAMN29132626; AB190, accession number SAMN29132627; AB378, accession number SAMN29132628; AB4616, accession number SAMN29132629; AB527, accession number SAMN29132630; AB925, accession number SAMN29132631; ABF246, accession number SAMN29132633; ABF489, accession number SAMN29132634; ABD267, accession number SAMN29132632).
