# Supplementary material for: Phenotypic and Genomic Characterization of Nine String-Positive Carbapenem-Resistant Acinetobacter baumannii Isolates from Israel
Source: Microbiol Spectr. 2023 Jan 31;11(2):e03002-22. doi: 10.1128/spectrum.03002-22 (PMC10100839; doi:10.1128/spectrum.03002-22)

Supplementary Table S1: String length in mm on a different agar plates.

	Blood		Chocolate		MH		Mackonkey		MDR	
	Av	Std	Av	Std	Av	Std	Av	Std	Av	Std
ABF489	23.60	2.88	31.20	2.59	7.80	1.92	20.80	3.11	25.60	2.70
AB527	24.20	2.39	8.00	2.12	10.20	1.48	22.00	1.58	10.60	1.52
AB4616	30.60	2.41	32.60	1.82	20.40	1.52	19.80	3.11	25.60	2.88
AB167	11.00	2.92	9.40	1.52	8.20	1.48	13.20	2.59	5.60	1.14
ABD267	10.40	2.07	1.20	1.30	7.40	1.82	1.00	1.41	0.80	1.10
AB925	10.60	1.67	7.40	1.95	9.00	2.74	10.60	2.30	21.80	2.39
ABF246	15.80	2.17	11.20	4.32	9.00	2.45	6.80	2.77	8.80	1.92
AB190	15.00	4.74	13.20	3.63	9.20	3.19	8.60	2.88	11.00	2.74
AB378	20.60	2.88	21.40	5.86	18.00	3.61	13.40	4.16	14.20	5.40

Supplementary Figure 1. Density gradient assay

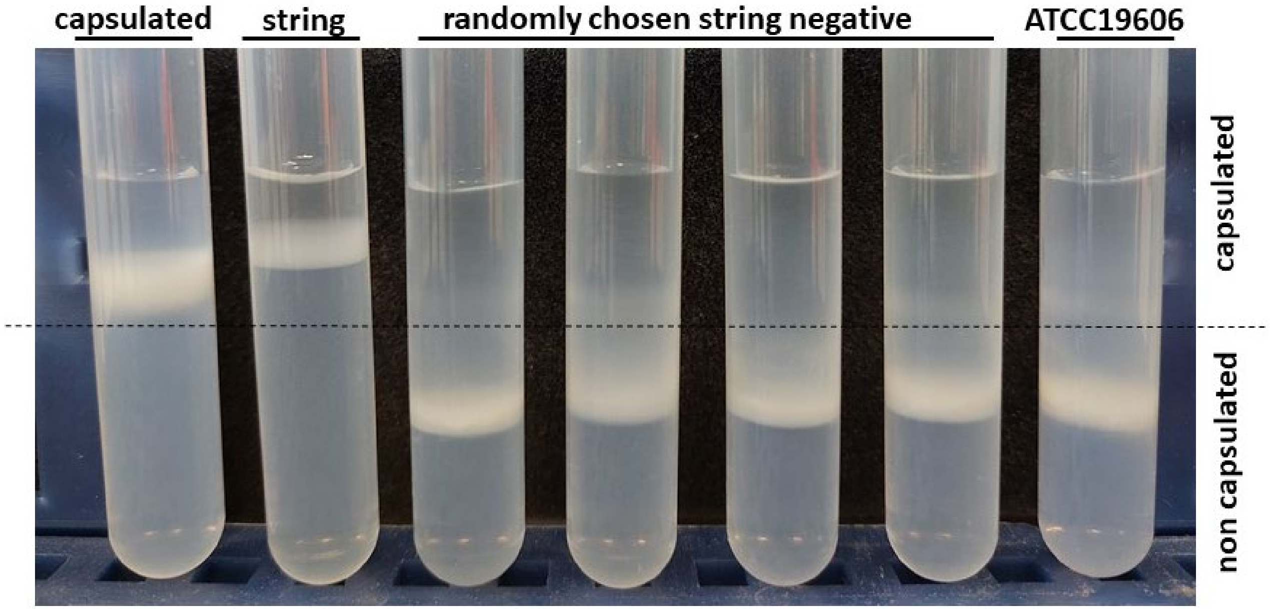

Supplementary Figure 1. Density gradient assay. The capsulated strain (Rakovitsky et al, 2021) migrated only to the top phase, as string positive isolates. The control strain ATCC 19606 migrated to the bottom phase, as control clinical non-string isolates, as thinly capsulated bacteria.

Supplementary Table S2. Individual motility for stCRAB strains.

	Diameter (mm)	SD	SEM
**AB527**	12.08	2.06	0.59
**AB378**	13.92	4.25	1.22
**AB190**	16.00	4.45	1.28
**ABD267**	22.50	6.97	2.01
**ABF489**	16.17	3.61	1.04
**ABF246**	12.67	2.70	0.78
**AB925**	15.25	4.35	1.25
**AB167**	5.75	1.21	0.35
**AB4616**	5.33	0.77	0.22
**ATCC**	5.41	1.08	0.31

Supplementary Figure S2. Phylogenetic tree of nine case string-positive isolates based on pangenomic analysis.

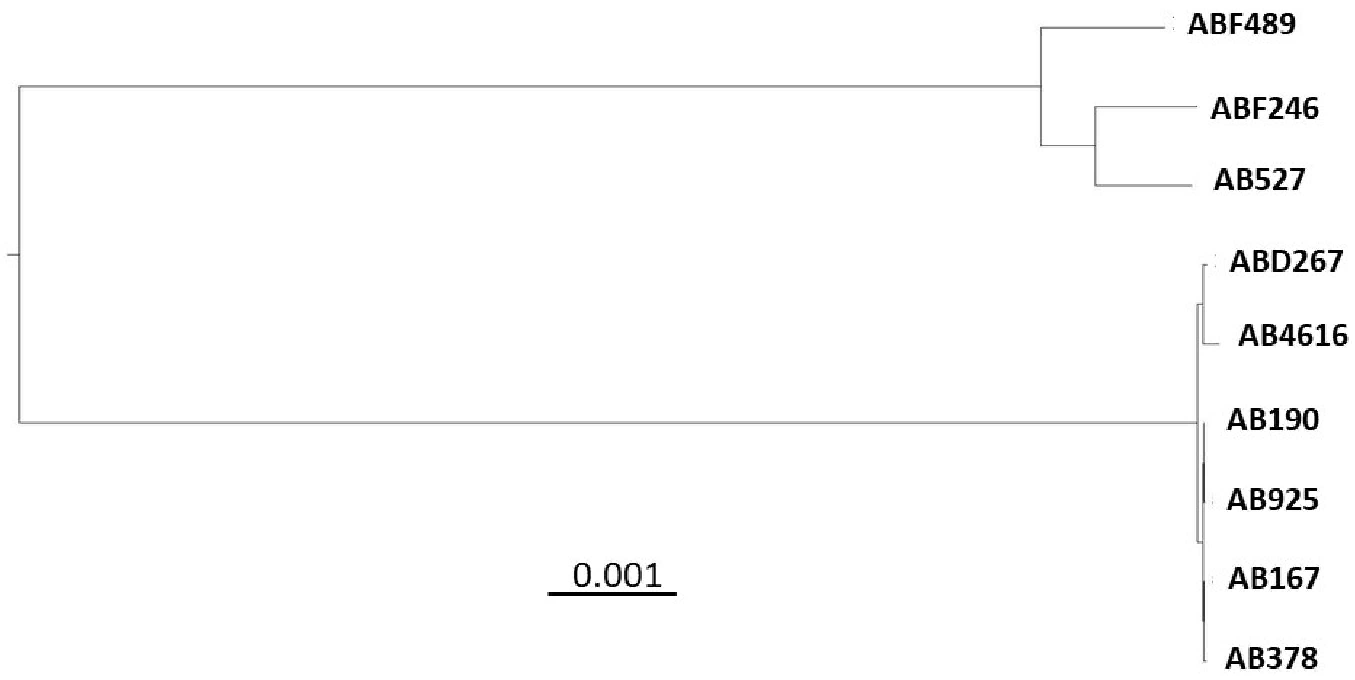

Supplementary Table S3: ANI results for pairwise strains analysis (nine string positive strains included)

AB527	AB489	AB246	ABD267	AB4616	AB925	AB378	AB190	AB167	
97.84	99.94	97.87	99.96	99.96	99.96	99.9	99.98		AB167
97.87	99.94	97.91	99.94	99.94	99.96	99.91		99.98	AB190
98.07	99.92	98.03	99.89	99.92	99.92		99.91	99.9	AB378
97.92	99.97	97.81	99.97	99.93		99.92	99.96	99.96	AB925
97.77	99.96	97.79	99.96		99.93	99.92	99.94	99.96	AB4616
97.84	99.94	97.87		99.96	99.97	99.89	99.94	99.96	ABD267
99.77	97.79		97.87	97.79	97.81	98.03	97.91	97.87	AB246
97.89		97.79	99.94	99.96	99.97	99.92	99.94	99.94	AB489
	97.89	99.77	97.84	97.77	97.92	98.07	97.87	97.84	AB527

Supplementary Table S4. SNP distance analysis. A core genome alignment was performed using Roary for three closely related phylogeneic groups; (1) ABF246 andABF305 (2) ABD258 and AB527 (3) ABE336, ABF155 and ABF489. Each group is comprised of one STRING positive and one or two STRING-negative isolates. SNPs were calculated using SNP-dists 0.8.2 program (https://github.com/tseemann/snp-dists)

Comparison	No. of SNPs
ABF246 vs. ABF305	152
ABD258 vs. AB527	141
ABE336 vs. ABF155	389
ABF155 vs. ABF489	51
ABF489 vs. ABE336	412

Supplementary Table S5. Accession number and ST types of genome used as a control in the study

Accession number	ST type
GCA_000214985.2	ST3
GCA_000241665.2	ST3
GCA_000278625.1	ST3
GCA_000278645.1	ST3
GCA_000286535.1	ST3
GCA_000305295.1	ST3
GCA_000309215.1	ST3
GCA_000368105.1	ST3
GCA_001612015.1	ST3
GCA_001612215.1	ST3
GCA_001950095.1	ST3
GCA_001950315.1	ST3
GCA_002150405.1	ST3
GCA_002183515.1	ST3
GCA_002416325.1	ST3
GCA_002416365.1	ST3
GCA_000367885.1	ST2
GCA_000189735.2	ST2
GCA_002416395.1	ST2
GCA_000513795.2	ST2
GCA_000188215.1	ST2
GCA_000509305.1	ST2
GCA_000509345.1	ST2
GCA_000359725.1	ST2
GCA_000299675.1	ST2
GCA_000417785.2	ST2
GCA_000302215.1	ST2
GCA_000307975.2	ST2

GCA_000189655.2		ST2
GCA_000419385.1		ST2
GCA_000804715.1		ST2
GCA_000805615.1		ST2
F223	SAMN16659894	ST3
D143	SAMN16659792	ST3
E409	SAMN16659854	ST3
D291	SAMN16659814	ST3
D292	SAMN16659815	ST3
F141	SAMN16659872	ST3
F155	SAMN16659882	ST2
F305	SAMN16659908	ST2
E336	SAMN16659849	ST2
F136	SAMN16659869	ST2
D258	SAMN16659806	ST2
D318	SAMN16659820	ST2

Supplementary Table S6: Resistome of the 9 case "string" isolates. + stands for presence of the gene in the genome.

			ST2			ST3					
**Antibiotic class**	**Function**	**Gene**	**AB527**	**ABF489**	**ABF246**	**AB167**	**AB190**	**AB378**	**AB925**	**AB4616**	**ABD267**
Aminoglycosides modifying enzymes	O-nucleotidyltransferases	ant (2')-Ia				+	+	+	+		+
	O-phosphotransferase	aph(3')-Ia	+	+	+						
		aph(3')-Via		+	+	+	+		+	+	
		aph(6)-Id	+	+	+			+			
		aph(3")-Ib	+	+	+			+			
β-Lactams	OXA-51-Like β-Lactamase	OXA-71				+	+	+	+	+	+
		OXA-66	+	+	+						
	Other OXA types β-Lactamase	OXA-23	+	+	+	+	+	+	+	+	+
	AmpC cephalosporinase	AmpC-6				+	+	+	+		
		AmpC-73	+	+	+					+	+
		AmpC-79								+	+
Sulfonamide	Dihydropteroate synthase	Sul1				+	+	+	+		+
		Sul2			+						
Tetracycline	Efflux major facilitator superfamily (MFS) transporter	Tet(A)				+	+	+	+		+
		Tet(B)	+	+	+			+			
Macrolide	ABC-type ribosomal protection protein	msr (E)	+	+	+			+			
	2' phosphotransferase	mph (E)	+	+	+			+			
Antiseptic resistance	Quaternary ammonium compound-resistance protein	QacE				+	+	+	+		+

Supplementary Table S7. Growth curve parameters for string positive strains in MH.

strain	lag_time	max_growth	doubling_time	growth_rates
ATCC	15.089	1.59	6.347	0.109
AB527	2.227	1.185	16.652	0.042
AB925	8.937	1.433	10.399	0.067
AB4616	7.923	1.089	20.069	0.035
ABF489	3.619	1.411	13.016	0.053
AB167	3.157	1.292	16.994	0.041
ABF246	3.781	1.703	8.519	0.081
ABD267	5.199	1.626	9.958	0.07
AB378	9.819	1.218	16.178	0.043
AB190	11.933	1.73	9.127	0.076

Supplementary Figure 3. Growth curve analysis for string positive isolates and ATCC19606.

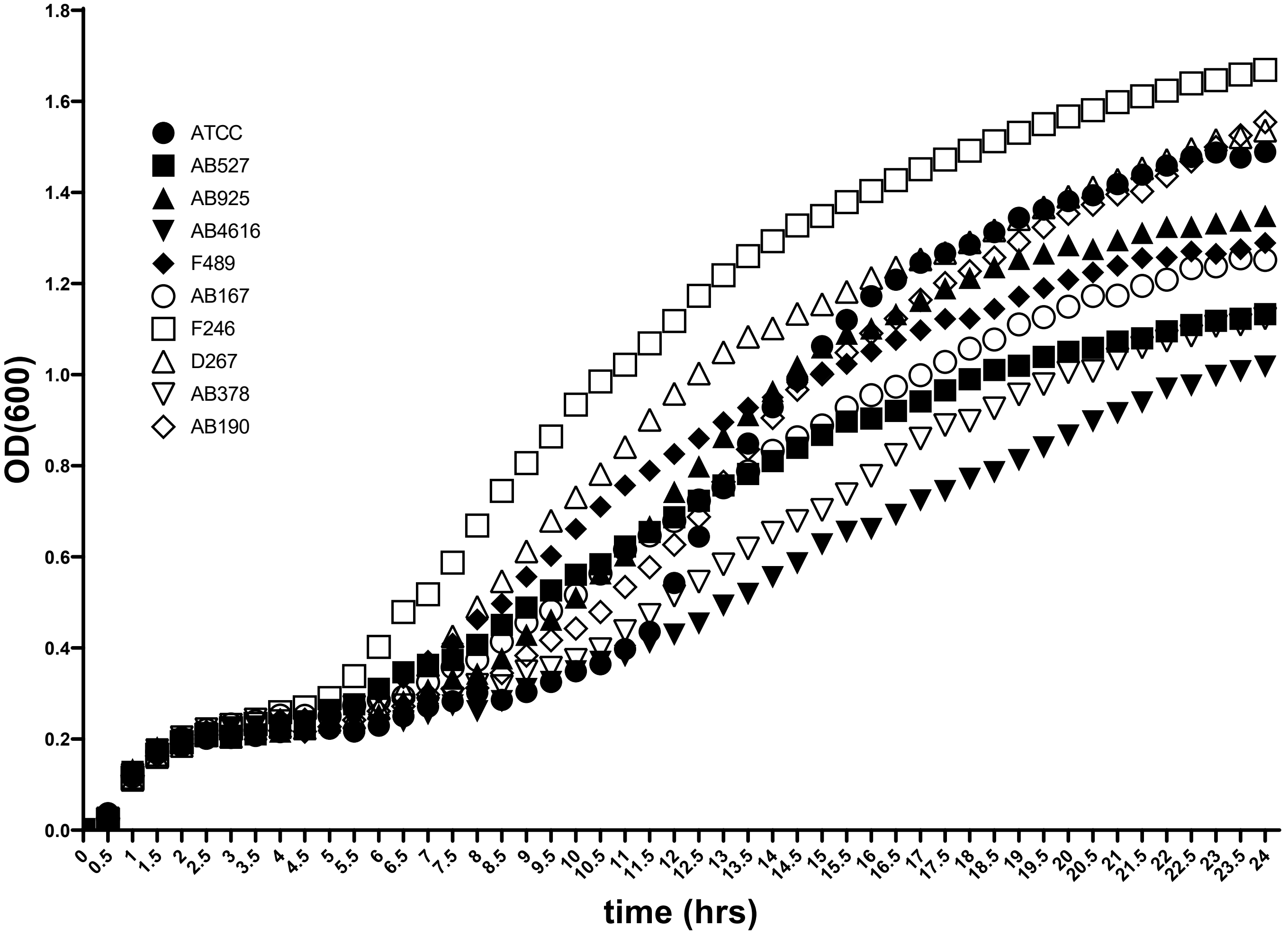

Supplement: Supplemental file 1 — Supplemental material. Download spectrum.03002-22-s0001.pdf, PDF file, 0.3 MB [file spectrum.03002-22-s0001.pdf]
